# Supplementary material for: Food disgust sensitivity predicts disease-preventing behaviour beyond the food domain in the COVID-19 pandemic in Germany
Source: PLoS One. 2021 Jul 14;16(7):e0254648. doi: 10.1371/journal.pone.0254648 (PMC8279350; doi:10.1371/journal.pone.0254648)
Supplement: S1 Appendix — (DOCX) [file pone.0254648.s001.docx]

**S1 Appendix**

Table 4: Items used to assess attitudes and behaviours related to the COVID-19 pandemic, including means (M), 95% confidence intervals (CI), and standard deviations (SD), N = 519

| # | Item | *M* | *SD* |
| --- | --- | --- | --- |
| 1 | I feel affected by the current situation regarding the spread of the coronavirus in Germany. | 4.28 | 1.53 |
| 2 | I feel threatened by the current situation regarding the spread of the coronavirus in Germany. | 3.62 | 1.59 |
| 3^a^ | I feel restricted in my personal freedom by the current situation regarding the spread of the coronavirus in Germany. | 4.01 | 1.69 |
| 4 | I consciously try not to touch my face with my hands. | 4.56 | 1.48 |
| 5 | I have built up an emergency supply of food. | 2.97 | 1.67 |
| 6 | I always wash my hands thoroughly with soap. | 5.34 | 1.04 |
| 7 | I have built up an emergency supply of toilet paper. | 2.44 | 1.64 |
| 8 | I avoid shaking hands with other people. | 5.49 | 1.09 |
| 9 | I bought disinfectant. | 3.68 | 2.08 |
| 10 | I am increasingly buying packed food (e.g. packed vegetables instead of vegetables in open sale). | 2.61 | 1.75 |
| 11^a^ | I got myself a protective mask. | 4.85 | 1.81 |
| 12 | I am increasingly buying long-life food (e.g. canned food, paste, …). | 3.04 | 1.68 |
| 13^ab^ | I meet regularly with people outside of my household. | 5.02 | 1.46 |
| 14 | I try to avoid public transport. | 4.99 | 1.66 |
| 15^ab^ | I use cloth handkerchiefs. | 4.49 | 2.04 |
| 16^a^ | I do not leave the house without an urgent reason. | 4.16 | 1.82 |
| 17 | I keep distance when I meet or come across people. | 5.44 | 1.02 |
| 18 | I try not to touch door handles any more. | 4.22 | 1.73 |
| 19 | When I show symptoms like fever or cough, I stay at home. | 5.51 | 1.04 |
| 20 | I am afraid of contracting the coronavirus. | 3.75 | 1.75 |
| 22 | I am afraid of contracting strangers with the coronavirus. | 3.91 | 1.68 |
| 21 | I am afraid of contracting my family and friends with the coronavirus. | 4.30 | 1.71 |
| 22 | I am afraid of contracting strangers with the coronavirus. | 3.56 | 1.77 |
| 23 | I am afraid of an overloading of the health care system. | 3.91 | 1.68 |
| 24 | I am afraid of no longer being able to get the products of daily use (food, toilet paper, …). | 2.79 | 1.65 |

*Note*. Statements were rated on a scale from 1 (*do not agree at all*) to 6 (*completely agree*). *^a^*: Items were excluded in the final version. ^b^: Items were recoded.
