# Supplementary material for: Food disgust sensitivity predicts disease-preventing behaviour beyond the food domain in the COVID-19 pandemic in Germany
Source: PLoS One. 2021 Jul 14;16(7):e0254648. doi: 10.1371/journal.pone.0254648 (PMC8279350; doi:10.1371/journal.pone.0254648)
Supplement: Attachment — Submitted filename: Reviewer_comments_R1.docx [file pone.0254648.s002.docx]

**PONE-D-21-12532**

We thank the editor and the reviewer for their considerate feedback. We have addressed all the comments and we have highlighted the changes made to the manuscript for your convenience. Below, we list the comments and explain how we have addressed them.

| **Editor’s comments** | **Authors’ reply** |
| --- | --- |
| I have now collected one review from one expert in the field. I was unable to find a second reviewer, but I am myself familiar with the topic of this manuscript, therefore I feel confident in making a decision with only one review. The reviewer is positive and suggests minor revision. I agree with the reviewer. Therefore, I would like to invite you to revise your work for Plos One. Needless to say that all the reviewer's comments should be addressed. | We thank the editor for the valuable and positive feedback. |
| Moreover, I would like to add a few more comments from my own reading of the paper, mainly regarding the literature review. Indeed, you report several interesting results that are in line with previous work, and so they should be discussed in this larger context: (i) You report a correlation between feeling of fears and preventative behaviour. This correlation has been reported also in other studies (Capraro & Barcelo, 2020; Dryhurst et al. 2020) | We agree with the editor that this was not sufficiently covered so far. We have added some more context as follows:  Introduction:  *Besides sociodemographic information such as sex (Capraro & Barcelo, 2020) and risk perception (Dryhurst et al., 2020), personal abilities, people’s intrinsic motivation and individual differences in psychological variables were found to play an important role in whether they follow virus-mitigating behaviour recommendations (Harper, Satchell, Fido, & Latzman, 2020; van Rooij et al., 2020).*  Methods:  *Given that previous research reported a relationship between risk perception and behaviour related to the pandemic (Dryhurst et al., 2020), we investigated self-reported behaviour and feelings regarding the COVID-19 pandemic in the second section (COVID-19 items).* |
| (ii) you find gender differences in preventative behavior. This was also reported previously (Capraro & Barcelo, 2020) | Thank you for this remark. We have added to the discussion as follows:  *In line with previous research (Brouard et al., 2020; Clark et al., 2020; van Rooij et al., 2020), we found that females showed more compliance with the COVID-19 behaviour recommendations than males. For instance, Capraro and Barcelo (2020) found that females were more likely to wear face covering than males. Our research shows that this effect also applies to other types of preventive behaviours such as staying at home or keeping some distance.* |
| (iii) This "perspective article" on what social and behavioural science can do to support pandemic response can be useful: Van Bavel et al. (2020).  I am looking forward for the revision. | Thank you for this remark. We included this research in the limitations and outlook section.  ... *As the pandemic progresses, it becomes harder to motivate the public to follow the behaviour recommendations. In this regard, social science can play an important role in supporting behaviour change (Bavel et al., 2020). Based on our results, we suggest that the use of disgust cues can be used as a measure to motivate preventive behaviour.* … |

| **Reviewer #1:** | **Authors’ reply** |
| --- | --- |
| The study investigated the link between food disgust sensitivity and an individual’s feelings, shopping behavior, and disease-preventive behavior related to the COVID-19 pandemic. The research question is interesting and the data are a valuable contribution to the field. Overall, it is an interesting and well-written manuscript. I enjoyed reading the submission very much. I have only minor comments to further improve the manuscript. | We thank the reviewer for this positive feedback and the helpful comments. |
| 1. My main concern: causation needs to be more carefully addressed. It seems, that authors assumed that increased food disgust sensitivity may predict higher levels of fear related to COVID-19. However, higher pathogen threat (i.e. fear related to COVID-19) may influence disgust sensitivity and pathogen avoidance behavior. It is worth citing some research that demonstrates experimental manipulations of pathogen threat might up-regulate disgust sensitivity (i.e., behavioral immune system processes). The following two papers may (or may not) be useful for some of the discussion on the link between disgust sensitivity and pathogen threat during COVID-19 pandemic: <https://doi.org/10.3389/fpsyg.2020.600761> <https://doi.org/10.3389/fpsyg.2021.622634>  The language throughout needs to be more cautious and authors should be more attentive to other potential causal scenarios. | We thank the reviewer for this remark. We worked through the manuscript, adapting the language, and adding more caution to the formulations. Importantly, we added more detail to the discussion:  *… Still, it needs to be acknowledged that correlation does not equal causation. The pandemic comes with an increased pathogen pressure, which in turn can up-regulate disgust sensitivity (Stevenson, Saluja, & Case, 2020). For instance, Stevenson et al. (2020) found that disgust levels assessed during the time of the pandemic were higher than the values reported before the pandemic. Similarly, we noticed that the average food disgust sensitivity in our sample was higher than the values reported in previous studies conducted in Germany before the pandemic (M = 3.84 [females] and M = 3.58 [males]; Egolf et al., 2019). These findings may serve as an indication that increased pathogen threat, as imposed through the pandemic, leads to an increase in disgust sensitivity. However, these interpretations should be further underpinned by additional research.* |
| 2. As a general rule, a full stop is not used at the end of a title. | We thank the reviewer for this remark. Full stop has been removed from the title. |
| 3. In the introduction, Authors write, “A common disgust response is avoidance, resulting in reduced pathogen contact and lower risk of infection (…) disgust is a pathogen-avoidance mechanism….” Consider specifying pathogen disgust here (reactiveness to contamination threats), since sexual and moral disgust do not appear to neutralize infection (see <https://doi.org/10.1037/a0030778>). | We agree with the reviewer that the pathogen disgust should be defined, especially as some of our argumentation builds on pathogen disgust (see comment below). Therefore, we have added the following definition to the introduction:  *Tybur, Lieberman, and Griskevicius (2009) identified three domains of disgust: (1) pathogen disgust, (2) sexual disgust and (3) moral disgust. Pathogen disgust can be seen as reactiveness to contamination threats and motivates avoidance of infectious microorganisms, resulting in reduced pathogen contact and lower risk of infection A common disgust response is avoidance, resulting in reduced pathogen contact and lower risk of infection (Curtis, 2011; Shook, Thomas, & Ford, 2019; van Overveld, de Jong, & Peters, 2010).* |
| 4. Perhaps a little more justification as to why, only food disgust sensitivity was measured. More space should be devoted in the introduction and methods to describing why you chose the measures you chose (the Food Disgust Scale), and why certain related measures (e.g., the Three Domain Disgust Scale, the Disgust Scale-Revised or sets of images associated with contagion risk) were not included. More transparency about your design decisions would be helpful for readers to evaluate the study's methods. | We thank the reviewer for this remark. We have added the following explanations.  Introduction:  *… We chose a food-specific measure of disgust sensitivity for three reasons. First, it has been demonstrated that there is a relationship between pathogen disgust and behaviours related to the pandemic (Gul et al., 2021). Given that we know that pathogen disgust and food disgust sensitivity are positively correlated (Egolf et al., 2019), we aimed to add to the evidence by investigating the role food disgust sensitivity plays in mitigating the effects of the pandemic. Second, the current pandemic has a huge impact on how we buy, prepare, and consume food. Therefore, we expect some conceptual overlap between food disgust sensitivity, hygiene, and behaviours related to the pandemic. Third, we aim to investigate whether food disgust sensitivity, due to its risk avoidance nature, explains variance beyond the domain of food. Given that enforcement of virus-mitigating measures is costly and public compliance cannot be taken for granted (Brouard et al., 2020), a better understanding of the mechanisms and motivations underlying virus-mitigating behaviours and attitudes can crucially contribute to the success of political measures aiming to contain the spread of the virus.*  Methods:  *The third and final section included a measure of disgust sensitivity. To the best of our knowledge, the FDS short is the only available disgust measure that specifically focuses on the domain of food. As a result, we used the 8-item short version of the Food Disgust Scale (FDS short, Hartmann & Siegrist, 2018) as a measure of food-specific disgust.* |
| 5. Any a priori decisions that factored into your sample size (rules of thumb, power analyses) should reported. | We thank the reviewer for pointing out that this information is missing. We have added it to the methods section:  *We aimed for at least 250 male and female participants each. Assuming small to medium effect sizes (d = .25), this sample size provides ample power (.80) to detect possible effects (α = .05) with a one-tailed test (Faul, Erdfelder, Lang, & Buchner, 2007). Similarly, a sample of 500 provides sufficient power to compare dependent Pearson’s correlations with common index (Faul, Erdfelder, Buchner, & Lang, 2009).* |
| 6. In affluent parts of the world it is possible to choose to avoid a pathogen if you think one may be present e.g. in expired food as other meals are available. In some environments people have no way to avoid similar warning signs – can it be assessed in future research directions? | We agree with the reviewer. We believe the environment is a very important aspect that needs to be investigated and discussed. We added it to the limitations and outlook section:  *… Given that similar constructs for food disgust sensitivity have been used cross-culturally (Ammann, Egolf, Hartmann, & Siegrist, 2020; Egolf et al., 2019), another interesting endeavour would be to test whether these relationships can be found across different countries. Not only have individuals been affected differently by the pandemic depending on their socio-demographic background (Martinez, Kopp, Lalive, Pichler, & Siegenthaler, 2021), but individual disgust sensitivity also depends strongly on the environment in which an individual lives (see Ammann, Hartmann, & Siegrist, 2019 for a discussion).* |
